# McLean’s Neuralgic Liniment

**Published:** 1862-07

**Authors:** 


McLean's Neuralgic Liniment.—Extract of belladonna, four grains; liquid ammonia, six fluid ounces; oil of turpentine and olive oil, of each half an ounce; tincture of opium, two ounces. Mix and apply during the paroxysms.—*Amer. Druggists' Circular.*

